# A comprehensive review of rehabilitation approaches for traumatic brain injury: efficacy and outcomes

**DOI:** 10.3389/fneur.2025.1608645

**Published:** 2025-06-13

**Authors:** Ye Shen, Linzhi Jiang, Junmei Lai, Jiahui Hu, Feng Liang, Xingru Zhang, Fangfang Ma

**Affiliations:** ^1^Department of Rehabilitation Medicine, Zhejiang Provincial People’s Hospital, People’s Hospital of Hangzhou Medical College, Hangzhou, China; ^2^Geriatric Medicine Center, Department of Geriatric Medicine, Department of Nursing, Zhejiang Provincial People’s Hospital (Affiliated People’s Hospital), Hangzhou Medical College, Hangzhou, Zhejiang, China

**Keywords:** traumatic brain injury, traumatic brain injury rehabilitation, cognitive therapy, telerehabilitation, medical trends

## Abstract

Traumatic Brain Injury (TBI), particularly in moderate-to-severe cases, remains a leading cause of long-term disability worldwide, affecting over 64 million individuals annually. Its complex and multifactorial nature demands an integrated, multidisciplinary rehabilitation approach to address the diverse physical, cognitive, behavioral, and psychosocial impairments that follow injury. We conducted a structured literature search using PubMed, Scopus, and Web of Science databases for suitable studies. This comprehensive review critically examines key rehabilitation strategies for TBI, including neuropsychological assessments, cognitive and neuroplasticity-based interventions, psychosocial support, and community reintegration through occupational therapy. The review emphasizes emerging technological innovations such as virtual reality, robotics, brain-computer interfaces, and tele-rehabilitation, which are expanding access to care and enhancing recovery outcomes. Furthermore, it also explores regenerative approaches, such as stem cell therapies and nanotechnology, highlighting their future potential in neurorehabilitation. Special attention is given to the importance of rigorous outcome evaluation, including standardized functional measures, neuropsychological testing, and advanced statistical methodologies to assess treatment efficacy and clinical significance. Patient-centered care is emphasized as a core element—rehabilitation plans are tailored to each individual’s cognitive profile, functional needs, and life goals. Studies show this approach leads to better outcomes in executive functioning, emotional wellbeing, and community reintegration. It identifies gaps in current research, such as the lack of longitudinal studies, predictors of individualized treatment success, cost–benefit evaluations, and strategies to manage comorbidities like PTSD. Thus, combining conventional and technology-assisted rehabilitation—guided by patient-centered strategies—can enhance recovery in moderate-to-severe TBI. Future research should focus on long-term effectiveness, cost-efficiency, and scalable personalized care models.

## 1 Introduction

TBI—including mild, moderate, and severe forms—is a significant global health issue. However, moderate-to-severe TBIs contribute disproportionately to long-term disability that impacts millions of individuals each year across the world. It leads to various physical, cognitive, and emotional difficulties, considerably reducing the quality of life of affected individuals (1, 2). Globally, estimates indicate that approximately 64–74 million individuals sustain a moderate-to-severe TBI annually, reflecting a substantial burden on healthcare systems worldwide (3). The broad spectrum of deficits arising from moderate-to-severe TBI includes impairments in physical functions, motor coordination, speech capabilities, and cognitive abilities. These impairments frequently result in enduring disabilities, significantly affecting everyday activities and interactions, and thereby requiring ongoing support and long-term rehabilitation (3).

Incidence is higher in males and varies by region; for example, Eastern Europe and Central Europe have among the highest age-standardized rates (4). Falls are the leading cause in most settings, and TBI incidence shows a bimodal age pattern (peaks in young children and the elderly) (4, 5). Low- and middle-income countries bear a disproportionately high burden (roughly three times higher incidence than high-income countries) due to factors like traffic injuries and limited healthcare resources (4).

The consequences of moderate-to-severe TBI extend far beyond the immediate injury period. Individuals often face long-term impairments such as difficulties in memory, attention, and organization, as well as increased frustration and emotional distress (2). Such impairments can negatively impact the individual’s ability to engage fully in social, occupational, and educational activities. The chronic nature of these difficulties emphasizes the necessity of comprehensive rehabilitation interventions that aim not merely at physical recovery but also cognitive and emotional improvement (2).

Rehabilitation following moderate-to-severe TBI is therefore a complex, multidisciplinary process. A multifaceted approach is critical, involving various therapeutic interventions customized to meet the individual needs and challenges of each patient. Addressing symptoms such as distractibility, memory impairments, disorganization, frustration, and feeling overwhelmed requires strategies that are general enough to provide broad benefits yet flexible enough to accommodate personal circumstances (1). Domain-general strategies, which focus on promoting current functioning, adaptation, and practical problem-solving, are especially beneficial in supporting patients’ long-term improvements and daily living skills.

Initially, management of moderate-to-severe TBI prioritizes stabilizing patients medically and preventing secondary injuries such as increased intracranial pressure, hypoxia, and infections. This acute phase is crucial; however, it represents only the initial step in a prolonged recovery journey (2). Once stabilized, the focus shifts significantly toward rehabilitation. Rehabilitation during this stage aims to maximize the patient’s functional recovery, improve independence, and enhance quality of life, both in the short and long term. Effective rehabilitation programs involve coordinated, integrated efforts from medical doctors, nurses, physiotherapists, occupational therapists, speech therapists, psychologists, and social workers. To improve care delivery, many centers now implement interdisciplinary case conferences, shared electronic health records, and role-defined care pathways to enhance communication, reduce redundancy, and ensure timely intervention across the team (6). Each professional brings a unique contribution to addressing the comprehensive needs of patients with moderate-to-severe TBI (6).

Neurorehabilitation specifically addresses deficits associated with brain injuries by focusing on both motor and cognitive functions. Novel approaches such as neurogaming and dual-task training are gaining traction for their ability to stimulate physical and cognitive pathways simultaneously. These interactive systems combine physical activity with cognitive challenges, improving engagement, motivation, and functional outcomes (7). Motor rehabilitation is essential in improving physical mobility, coordination, balance, and strength. Such rehabilitation enables individuals to regain independence in daily activities, promoting self-sufficiency and improving overall life satisfaction. Concurrently, cognitive rehabilitation addresses impairments in memory, attention, executive functioning, and problem-solving skills, which are commonly compromised after moderate-to-severe TBI (3). Cognitive rehabilitation employs structured training and practical activities designed to enhance cognitive performance, thereby facilitating better integration into daily life and social contexts.

Moreover, the development of personalized rehabilitation strategies is essential for effective treatment outcomes. Rehabilitation plans must be individually tailored, taking into account each patient’s specific condition, severity of the injury, personal goals, and available support networks. An interdisciplinary team collaboratively develops and implements these rehabilitation strategies, ensuring comprehensive care through ongoing assessment, adaptation, and reinforcement of therapeutic interventions (6).

The growing incidence of severe TBI globally underlines the urgent need for effective, accessible, and innovative rehabilitation approaches. As the prevalence continues to rise, the economic and societal impacts also escalate, highlighting the necessity of advancing rehabilitation methodologies to improve outcomes for individuals and reduce the societal burden of moderate-to-severe TBI (2). Recent advancements in technology have notably contributed to expanding the reach and effectiveness of rehabilitation services. Telemedicine, in particular, has emerged as a valuable approach, effectively bridging the gap between specialized care providers and patients in remote or underserved regions (8). However, global implementation remains uneven due to barriers such as limited internet connectivity, low digital literacy among patients and providers, infrastructure disparities, and difficulties in ensuring long-term adherence in rural or resource-constrained settings (8, 9).

Telemedicine facilitates access to expert healthcare services, allowing patients in geographically isolated areas to receive timely, specialized interventions without the need for extensive travel. For instance, the integration of telemedicine in intensive care and trauma management settings (tele-ICU) significantly enhances patient care quality, providing critical support even in peripheral or resource-limited healthcare centers (8). Despite its promise, remote rehabilitation faces challenges such as poor internet infrastructure, digital illiteracy among older patients, and reduced engagement in unsupervised settings. Adherence can also be compromised by low motivation and lack of physical presence, particularly in rural or low-resource settings (8, 10), ensuring equitable healthcare distribution and broadening the scope and efficacy of moderate-to-severe TBI rehabilitation globally.

This review aims to present a thorough overview of current state-of-the-art rehabilitation strategies for moderate-to-severe TBI, critically evaluating their effectiveness and impact on patient recovery. Emphasis is placed on the importance of standardized care practices within dynamic clinical settings, ultimately seeking to enhance rehabilitation outcomes, patient satisfaction, and overall quality of life for those affected by moderate-to-severe TBI.

## 2 Current rehabilitation approaches for traumatic brain injury

Rehabilitation after moderate-to-severe traumatic brain injury (TBI) is a complex process that requires a clear understanding of the different causes and effects of the injury. Studies included in this review typically enrolled adults with moderate-to-severe TBI, using Glasgow Coma Scale scores, post-traumatic amnesia duration, or neuroimaging findings as inclusion criteria. Common exclusions included pre-existing psychiatric illness, progressive neurological conditions, and severe aphasia or physical limitations that hinder therapy participation (11–13). Variations in age, sex, injury mechanism, and comorbidities (e.g., PTSD, depression, cardiovascular disease) influenced intervention responsiveness, highlighting the need for stratified analyses and caution in generalizing findings across populations (1, 14). The foundation of successful rehabilitation is built on three main strategies: restitutional (restoring lost functions), compensatory (finding ways to work around deficits), and adaptive (adjusting to new ways of living). Selection of rehabilitation approaches was often based on the nature of cognitive, behavioral, or motor deficits observed in the patient. For example, neuropsychological therapies were prioritized for executive function deficits, while motor-focused rehabilitation was used for movement impairments. Interdisciplinary teams typically matched therapy types to target domains identified during baseline assessments (6, 15). Since TBI can lead to many types of difficulties—such as problems with thinking, emotions, behavior, and social interactions—rehabilitation needs to be personalized to address each of these areas effectively (16).

To support better outcomes, rehabilitation often includes medications alongside behavioral and cognitive therapies. This combination is important for managing the psychiatric symptoms that commonly occur after a TBI, such as depression or anxiety (6). Several cognitive rehabilitation techniques have shown good results (Table 1). These include direct cognitive training, teaching strategies to solve problems, metacognitive training (which helps patients think about their thinking), communication skills training, and using external memory aids. Even though these techniques are effective, more research across different cultures is needed to better understand how well they work in various populations (17).

**Table 1:** An overview of key rehabilitation strategies for moderate-to-severe TBI, outlining the primary techniques used, the specific functional deficits targeted, and the reported clinical outcomes.

Approach	Key techniques/interventions	Targeted deficits	Reported outcomes	References
Neuropsychology	Neuropsychological assessment, EEG neurofeedback, cognitive profiling	Cognitive (attention, memory, EF), emotional and behavioral issues	Improved cognitive mapping, tailored interventions, neurofeedback led to significant improvement in executive function and attention over 9 months	Jarrahi et al. (25), Tian et al. (26), and Santos et al. (28)
Cognitive rehabilitation	Restorative and compensatory strategies, attention/memory training, executive function support, computer-assisted programs	Attention, memory, executive function, information processing	Executive function gains (Hedges’ g = 0.48, *p* < 0.01); processing speed improved by 15–20% in multiple trials	Bogdanova et al. (11), Hallock et al. (83), and Parasuraman and Mathew (93)
Neuroplasticity-based rehabilitation	Task-specific training, constraint-induced movement therapy, brain stimulation, cranioplasty	Motor and cognitive impairments post-injury	Task-specific rehab enhanced cortical reorganization; early intensive therapy improved functional gains and brain remodeling (e.g., tDCS and CIMT combined)	Nudo et al. (21), Su et al. (22), and Lecani et al. (34)
Technological rehabilitation (VR, Robotics, AI)	Virtual reality training, robotics, brain-computer interfaces (BCI), AI-driven diagnostics and therapy	Motor dysfunction, cognitive challenges, engagement barriers	VR improved attention, executive function (20–30% gains) in patients with moderate-to-severe TBI; robotics improved gait speed and arm function (*p* < 0.05); AI-enhanced robots reduced depression (↓17.5 pts. on BDI-II, *p* < 0.001) and improved QoL (+1.5 EQ-5D)	De Luca et al. (90), Maggio et al. (24), and Andrei et al. (88)
Behavioral and psychosocial rehabilitation	Cognitive Behavioral Therapy (CBT), group therapy, contingency management, emotional regulation	Aggression, impulsivity, depression, PTSD, poor social skills	CBT and multimodal therapy reduced aggression and psychiatric symptoms, improved coping skills and social function	Cattelani et al. (62), Gilboa (63), and Teichner et al. (66)
Community-based rehabilitation and occupational therapy	Vocational training, job placement, ADL support, home-based programs, interdisciplinary support	Impaired community participation, work reintegration, daily functioning	Increased job success, independence, and social participation; cost-effective model using local support networks	Kim and Colantonio (19) and Powell et al. (20)
Attention and communication training	Attention process training, speech and language therapy, pragmatic language training	Aphasia, dysarthria, attention deficits, social communication problems	Significant improvements in attention and communication skills; improved pragmatic skills and social interactions	Tobar-Fredes and Salas (68) and Michel and Mateer (67)
Telerehabilitation and remote technologies	Video conferencing, mobile apps, wearable devices, tele-monitoring, tele-ICU	Limited access to care, cognitive/motor deficits, rural healthcare gaps	Improved functional independence (*p* < 0.001); executive function (FAB scores, *p* < 0.001); symptom self-management (SMD = 0.22); reduced anxiety (effect size ≈ 0.85)	Calabrò et al. (12), Jeon et al. (87), and Suarilah et al. (10)

References indicate the supporting evidence for each approach.

Art therapy has also become an important part of neurorehabilitation programs. It provides a flexible and creative way to meet the specific needs of each patient while following established brain injury rehabilitation principles (18). Community reintegration—defined as resuming participation in work, family life, and social activities—is a critical outcome. It is measured through tools like the Community Integration Questionnaire. Success is influenced by patient motivation, social support, access to vocational training, and tailored rehabilitation programs (3, 19, 20). Recent trials have also measured the role of neuroplasticity in driving recovery by tracking changes in brain structure and function over time using imaging or standardized cognitive tests. Longitudinal outcomes were assessed using tools like the Functional Independence Measure or neuropsychological batteries to evaluate sustained improvements (21, 22). Both laboratory and clinical studies are exploring how the injury continues to affect people over time, and how new treatments might address these lasting effects (23). Today, common treatments include medications, physical therapy, and sometimes surgery. However, while helpful, these options are often not enough to fully return patients to their previous level of functioning. Innovative modalities like VR, robotics, and AI were prioritized in studies due to their ability to enhance engagement, provide task repetition, and offer real-time feedback—benefits not always achievable through traditional methods. However, their comparative effectiveness varied, with some trials showing VR to outperform therapist-led interventions, while others found no added benefit over conventional therapy (12, 24).

### 2.1 Role of neuropsychology in TBI rehabilitation

Neuropsychological assessment plays an important role in understanding how traumatic brain injury (TBI) affects a person’s thinking, emotions, and behavior. This kind of evaluation helps in creating focused rehabilitation plans based on the individual’s needs (25). A full neuropsychological assessment looks at different areas of cognitive function, such as attention, memory, problem-solving, language, and visuospatial skills. It also includes examining emotional health, mood, and personality traits (26). These tests provide detailed information about the type and level of cognitive challenges a patient may have, which helps healthcare providers choose the most suitable therapies and track progress over time.

**Figure 1:**
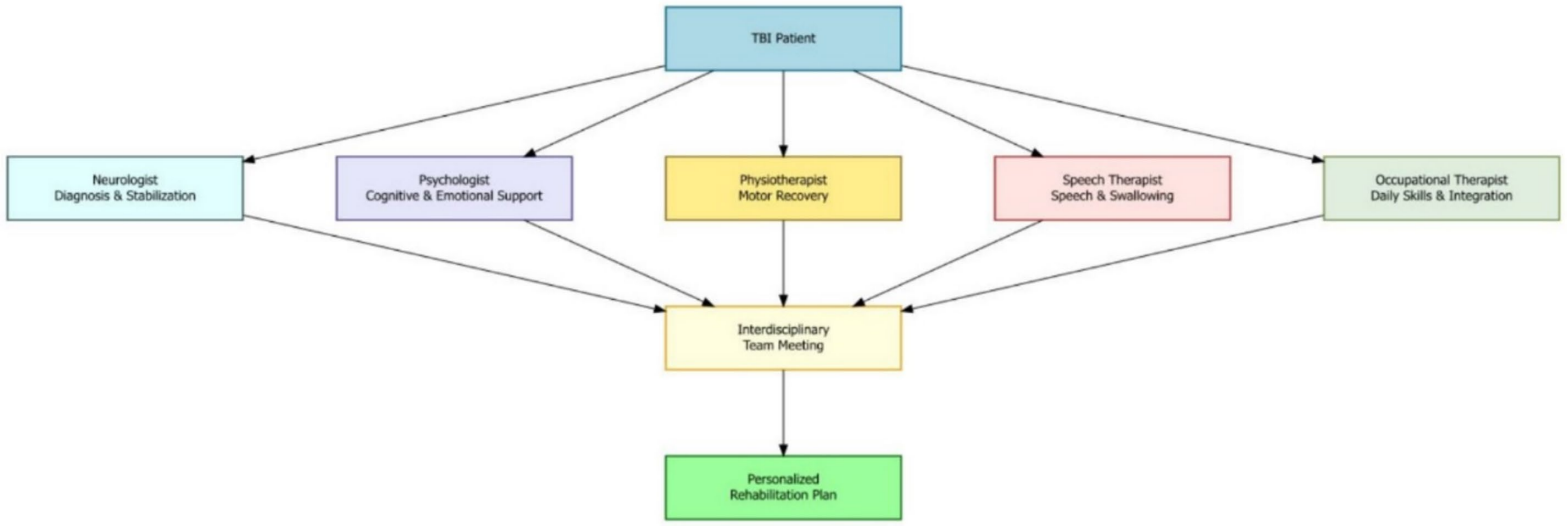
Multidisciplinary team model for moderate-to-severe TBI rehabilitation and roles of key specialists which are coordinated to develop a personalized rehabilitation plan through interdisciplinary collaboration.

In addition to guiding treatment, neuropsychological assessments help identify a patient’s cognitive strengths and weaknesses. This knowledge can be used to design strategies that use the patient’s strengths to support areas of weakness, improving their chances of recovery. These evaluations can also distinguish whether the cognitive problems are directly caused by TBI or are influenced by other factors, such as previous learning difficulties, mental health issues, or substance use (Figure 1).

One promising tool in this area is neurofeedback. In this method, patients receive real-time feedback about their brain activity through visual or sound signals. This approach has shown potential in reducing some TBI symptoms and may even be usable at home (27). For example, in one severe TBI case, a 25-year-old male with attention, executive, and visuospatial difficulties improved significantly after 9 months of treatment with EEG neurofeedback and psychosocial support (28).

Understanding a patient’s full cognitive and emotional profile through neuropsychological assessment allows clinicians to build rehabilitation plans that directly match each person’s needs and goals. Since psychiatric symptoms such as problems in memory, perception, language, and intelligence are common after moderate-to-severe TBI, these assessments are vital (29).

Neuropsychology is therefore essential to moderate-to-severe TBI care. It focuses on finding and understanding thinking and behavioral difficulties to design personalized treatment plans. These evaluations help identify challenges in attention, memory, and planning, allowing for more effective support in everyday tasks and social situations (113). Because thinking, emotion, and behavior are closely connected, a team approach that includes different specialists is often needed. This allows for well-rounded care that addresses all aspects of recovery. Personalized programs that target specific issues can greatly improve quality of life for moderate-to-severe TBI patients. Some survivors, referred to as the “walking wounded,” may have hidden but serious challenges that require thoughtful and specialized care (29). Early and correct identification of cognitive and emotional issues is key to providing the right care and achieving better long-term recovery for those living with moderate-to-severe TBI.

### 2.2 Neuroplasticity-based rehabilitation strategies

**Figure 2:**
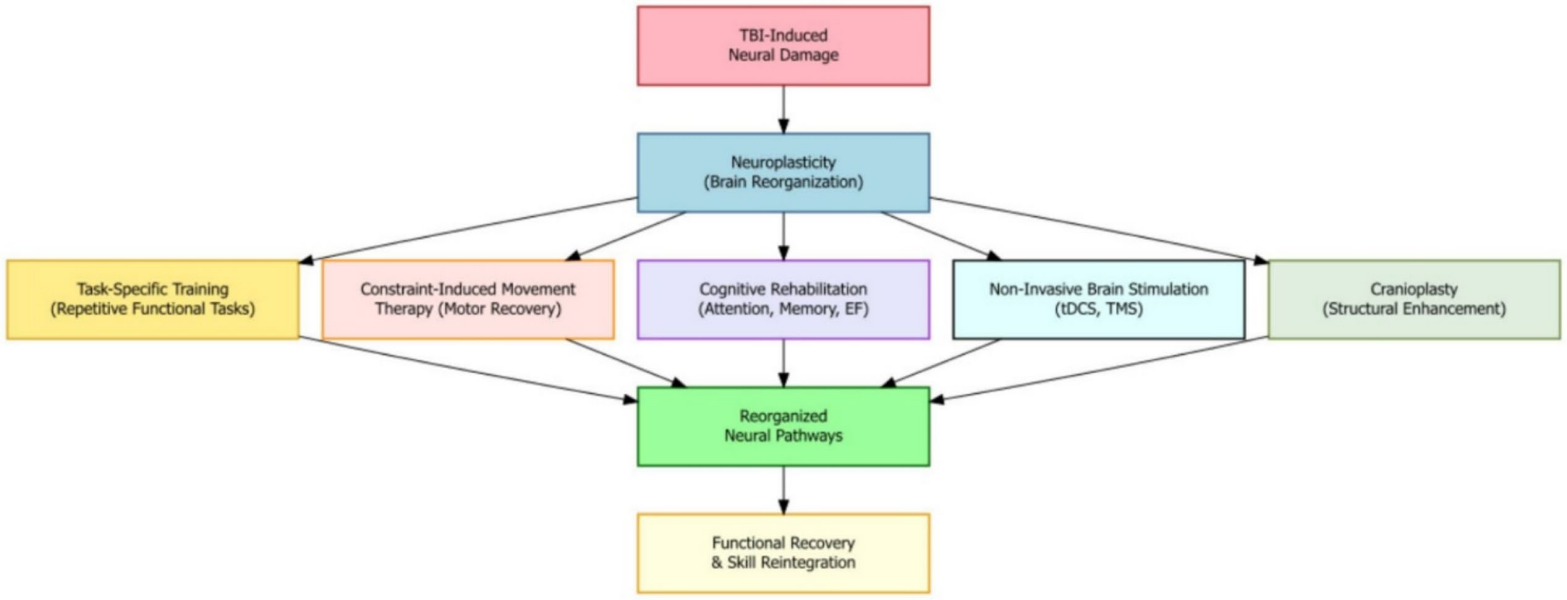
Neuroplasticity mechanisms in moderate-to-severe TBI recovery where therapeutic interventions reorganize brain pathways to promote functional recovery and skill reintegration.

Harnessing the brain’s natural ability to change and adapt—known as neuroplasticity—is a key part of recovery after moderate-to-severe traumatic brain injury (TBI). Neuroplasticity is the brain’s ability to reorganize its structure and function in response to learning, experiences, or injury. Many rehabilitation strategies are designed to take advantage of this ability to support healing, reorganize brain pathways, and help the brain adapt after injury. For example, task-specific training, which involves repeating functional activities, has been shown to create positive changes in motor and cognitive areas of the brain, helping patients regain skills and improve performance. Another technique, constraint-induced movement therapy—often used for stroke—can also help severe TBI patients with motor problems by encouraging the use of the weaker limb and supporting motor function recovery (Figure 2).

Cognitive rehabilitation methods, such as training for attention, memory, and executive function, work by encouraging neuroplastic changes in related brain areas. These approaches can improve thinking skills and overall brain function. New research is giving us better ways to study how the brain changes after injury, helping speed up both basic science and therapy development (21). In some cases, combining rehabilitation with cranioplasty—a surgical repair of the skull—has shown to further boost cognitive improvement (22). Medications that enhance brain chemicals or support growth factors may also improve neuroplasticity and support recovery. The timing of rehabilitation is very important, as the brain’s ability to change varies at different stages after injury. Research suggests that starting rehabilitation early and doing it intensively can be especially helpful.

By understanding how neuroplasticity works and using treatments supported by research, doctors and therapists can help people with TBI recover better and enjoy a higher quality of life (21). Physical therapy and other experiences can improve brain function and structure, but we still need more research to understand how this works in children and adults with conditions like cerebral palsy (30). Constraint-induced movement therapy and similar approaches encourage the brain to develop new pathways to make up for damaged ones.

However, understanding how and why rehabilitation methods work is still a big challenge (31). After a stroke, the brain begins to compensate for damage by adjusting its function (32). Learning more about how this process works is key to creating the best rehabilitation programs and promoting recovery (33). Non-invasive brain stimulation tools like transcranial magnetic stimulation and transcranial direct current stimulation (tDCS) are also showing promise in helping brain activity and supporting neuroplasticity in moderate-to-severe TBI rehabilitation (34). Still, more research is needed to find the best ways to use these tools and determine which patients benefit most.

Overall, neuroplasticity—the brain’s ability to adapt—is central to helping people recover from moderate-to-severe TBI. Techniques like constraint-induced movement therapy and virtual reality (VR) use this adaptability to improve both motor and thinking abilities. These methods help brain connections become stronger and more flexible, which can lead to better recovery results (114, 115). Using these therapies alongside new technologies like brain stimulation may further improve recovery and quality of life for moderate-to-severe TBI patients.

### 2.3 Cognitive rehabilitation approaches

Cognitive rehabilitation helps individuals with *mild-to-moderate traumatic brain injury (TBI)* manage problems with attention, memory, and executive function using both restorative and compensatory techniques. Restorative strategies are designed to improve damaged cognitive functions through repeated exercises and tasks, while compensatory methods teach new ways to work around these difficulties. These approaches have been shown to improve daily functioning and overall quality of life. Cognitive problems caused by severe TBI—such as trouble focusing, forgetting information, poor decision-making, or slow thinking—can seriously impact a person’s ability to work, study, maintain relationships, or complete everyday activities (Table 2).

**Table 2:** Cognitive rehabilitation techniques used in cognitive rehabilitation for individuals with mild to severe TBI, distinguishing between restorative and compensatory strategies.

Technique	Type (restorative/compensatory)	Targeted function	Example methods	Evidence/effectiveness
Attention training	Restorative	Sustained, selective, and divided attention	Computer-based attention drills, paper-pencil tasks	Shown to improve focus and task persistence (35)
Memory retraining	Restorative	Encoding, storage, and retrieval of information	Spaced retrieval, errorless learning, mnemonics	Effective in TBI recovery; boosts recall and daily task performance (36)
Executive function training	Restorative	Planning, organization, problem-solving, decision-making	Task analysis, strategy games, real-life problem solving	Improves real-world task planning and problem-solving (15)
Metacognitive training	Restorative	Self-monitoring, error recognition, adaptive thinking	Goal Management Training, self-questioning techniques	Enhances self-regulation and transfer of learning (36)
Use of external memory aids	Compensatory	Support for memory deficits in daily life	Planners, alarms, notes, digital reminders	Promotes independence and reduces caregiver burden (38)
Computer-assisted cognitive training	Restorative	Processing speed, attention, reasoning	Stepwise digital exercises targeting multiple domains	Builds confidence and cognitive speed (35)
Group cognitive rehabilitation	Compensatory	Cognitive and social functioning	Interactive cognitive-behavioral sessions with peers	Supports motivation, engagement, and social cognition (41)
Cognitive education for patients and families	Compensatory	Understanding cognitive deficits and managing them in daily life	Psychoeducation sessions, caregiver training	Improves coping strategies and daily task management (38)

It includes the cognitive functions targeted, example methods employed in therapy, and evidence of effectiveness drawn from published sources.

Rehabilitation usually combines restorative and compensatory strategies. For example, attention training might include tasks that strengthen focused, selective, or divided attention. Memory training can use tools like spaced retrieval, errorless learning, or memory tricks to help with remembering. Executive function training helps with planning, organizing, problem-solving, and making decisions. Computer-based programs can also help restore cognitive processing speed and accuracy, which often boosts the patient’s confidence (35). Overall, cognitive retraining supports skills like perception, focus, understanding, learning, remembering, and reasoning (36).

Though cognitive rehabilitation helps reduce symptoms, long-term and significant improvements are still hard to achieve (37). Educating patients and their families about the nature of cognitive problems and how to manage them is also an important part of the process (38). In some cases, cognitive training is also used to enhance thinking skills in people without any impairments (39).

The environment and method of therapy delivery also influence success, especially for people with conditions like multiple sclerosis (40). Group therapy has also been shown to benefit people with cognitive issues (41). Because cognitive impairments affect quality of life (42, 43), therapy must be tailored to each person’s needs and goals. This can be done using manual exercises or computer-based programs that provide engaging, multi-sensory tasks to boost motivation (36).

Research shows that repeated practice can improve attention, memory, and decision-making skills (44), and that varying training tasks can help with brain recovery (45). New tools like virtual reality and serious games are also being used to support cognitive recovery by creating immersive and realistic environments for practicing these skills.

### 2.4 Technological innovations in TBI rehabilitation

Innovative technologies like virtual reality (VR), robotics, and telerehabilitation are bringing major changes to how moderate-to-severe traumatic brain injury (TBI) is treated. VR systems, whether designed for the public or built specifically for healthcare, have shown success in improving both movement and thinking skills in patients with *moderate-to-severe TBI*. These tools create immersive environments that encourage task-based practice, helping the brain to recover through neuroplasticity (90, 116). VR also keeps patients engaged and provides the high repetition needed for meaningful recovery (46). Telerehabilitation makes it easier for patients to access treatment remotely, especially in areas with fewer healthcare services. It can improve outcomes by offering assessments and therapy at a distance (Table 3).

**Table 3:** Technological tools in moderate-to-severe TBI rehabilitation presenting a comparative overview of major technological interventions in traumatic brain injury rehabilitation, which highlights their functions, unique advantages, usability in clinical and home settings, and supporting references from current literature.

Technology	Function/application	Advantages	Accessibility (clinic/home-based)	References
Virtual reality (VR)	Motor and cognitive training through immersive, interactive simulations	Enhances motivation, allows task repetition, simulates real-life tasks, provides real-time feedback	Both clinic and home (via headsets and guided software)	De Luca et al. (90), Pietrzak et al. (116), and Allegue et al. (46)
Robotics	Delivering repetitive, high-intensity motor training for movement restoration	Precise, consistent movement practice; enables feedback and progress monitoring	Primarily clinic-based; some home versions under development	Barría et al. (119), Lazarević and Živković (98), and Villa et al. (108)
Brain-computer interface (BCI)	Enhancing motor control and communication via direct brain-device interaction	Promotes neural plasticity; supports patients with severe motor deficits	Mostly research/clinic-based with limited home usability	Simon et al. (51) and Drigas and Sideraki (48)
Artificial intelligence (AI)	Supporting diagnosis, treatment planning, progress tracking, and personalized care	Improves accuracy and speed of diagnosis; facilitates personalized rehabilitation pathways	Clinic-based systems; emerging integration into telehealth platforms	Zhu et al. (47) and Khalid et al. (39)
Telerehabilitation	Remote therapy delivery, monitoring, assessment, and patient support	Increases access to care, reduces travel/costs, enables therapy in underserved areas	Home-based and clinic-integrated	Algarni et al. (9) and Haranath et al. (8)
Wearable devices and mobile apps	Real-time tracking of physical/cognitive progress and providing adaptive feedback	Portable, user-friendly, supports daily therapy routines, encourages self-management	Primarily home-based with growing integration into clinical systems	Edwards et al. (117) and Qian et al. (61)

**Figure 3:**
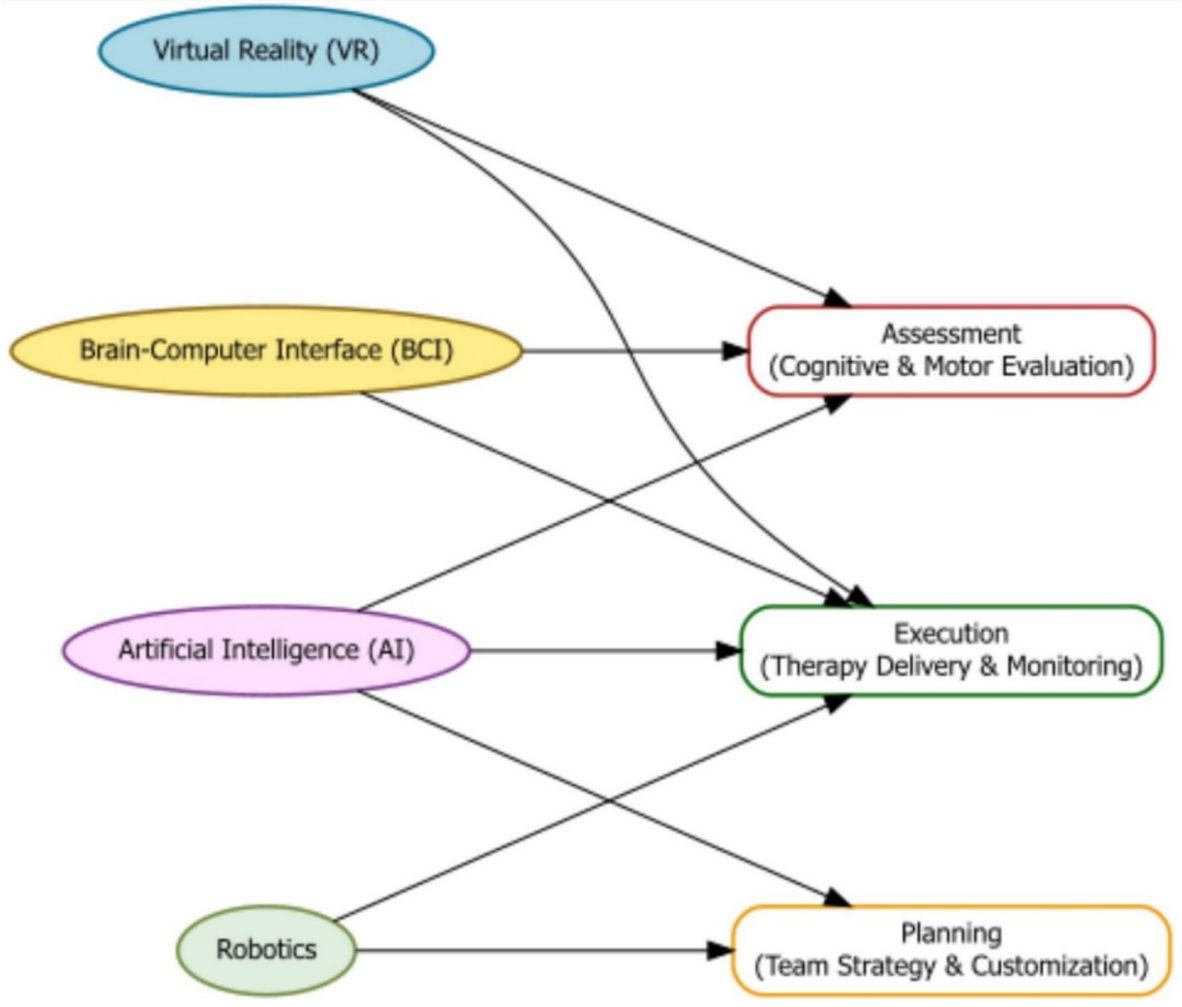
Flowchart illustrating how emerging technologies—Virtual Reality (VR), Brain-Computer Interfaces (BCI), Artificial Intelligence (AI), and Robotics—contribute to the assessment, planning, and execution phases of moderate-to-severe TBI rehabilitation.

Artificial intelligence (AI) is becoming a powerful tool in moderate-to-severe TBI care. It can support diagnosis, plan treatments, monitor progress, and provide personalized therapies (47). VR and brain-computer interface (BCI) technologies can make rehabilitation more interactive and effective (48). Robotics, BCI devices, and VR are being used to help patients regain motor and cognitive abilities (49–51). VR systems provide controlled, safe environments for therapy, allowing patients to practice daily tasks or simulate real-world challenges that are otherwise unsafe or difficult in clinics (52–54) (Figure 3).

Some VR programs can even be used at home, guiding patients through exercises tailored to their treatment plan (55). VR offers unique benefits by combining engaging, interactive training with repetitive practice in a safe space (56). It allows therapists to adjust task difficulty and provide sensory feedback (57). VR can also be used in research to study safety and attention through simulations (58). It’s also being used in elderly care homes, such as for Alzheimer’s patients, to help improve their condition (59). In fact, VR rehab programs are gaining popularity, with preliminary evidence suggesting they may be more effective than some traditional interventions in specific contexts (60).

Telerehabilitation offers benefits like lower costs, less travel time, and better access to services—particularly for people living in remote areas. It can be used for diagnosis, therapy, prevention, advice, and consultation (9). VR-based exercise has been found to help with physical health, mental wellbeing, and recovery when compared to regular exercise programs (61). As VR becomes more available and affordable, its role in moderate-to-severe TBI care is expected to grow, improving outcomes and patient quality of life (53, 58). Robotic rehabilitation also offers benefits by delivering consistent, high intensity training sessions and giving real-time feedback. These robotic systems are especially helpful in improving movement and helping patients build strength and coordination.

### 2.5 Community integration and occupational therapy

Occupational therapy plays an important role in helping people with moderate-to-severe traumatic brain injury (TBI) return to everyday life in their communities. These interventions focus on daily living tasks and social participation, and they have been shown to improve outcomes for people with TBI. Programs that involve multiple healthcare professionals and are centered around the patient’s goals—especially those based in the community—are key to helping people reintegrate successfully (19, 20).

Rejoining the community after a moderate-to-severe TBI involves physical, mental, and social challenges. Because of this, a complete and balanced approach is needed to support the different difficulties people face when returning to community life. Community-based rehabilitation programs aim to help individuals become more independent, improve their quality of life, and participate more fully in society. These programs may include job training, help finding employment, and ongoing support in the workplace, which can increase job success and financial independence.

Community-based rehabilitation is also a practical and cost-effective solution, especially in developing countries. It often uses home-based activities and makes use of local resources, making care more accessible. The main goals of these programs are to support independent living, help individuals return to work, and promote full participation in community life.

Vocational and community-based programs are especially effective because they provide personalized support to deal with the cognitive, emotional, and social difficulties that come with moderate-to-severe TBI. They also offer tools and guidance that help people reenter the workforce and maintain social connections. These interventions make a meaningful difference in how well people with TBI reintegrate into society and rebuild their lives.

### 2.6 Behavioral and psychosocial rehabilitation

Addressing behavioral and psychosocial challenges is a critical part of recovery for individuals with moderate-to-severe traumatic brain injury (TBI). Evidence-based treatments like cognitive-behavioral therapy (CBT) and applied behavior analysis are commonly used, while more comprehensive programs show the strongest improvements in emotional and social functioning (62). Psychosocial care, particularly CBT, group therapy, and peer mentoring, significantly improves emotional regulation, reduces depression and aggression, and enhances social reintegration. These therapies also support families by reducing caregiver stress and improving the rehabilitation environment (62, 63).

Psychosocial rehabilitation targets emotional, behavioral, and social problems that can occur after TBI, supporting better mental health and improved interactions with others (63). After the physical recovery stage, it is important to deal with the cognitive, emotional, and behavioral difficulties that can continue to affect daily life (16). In many cases, a mix of behavioral therapies and medications is the most effective way to manage these complex problems (6).

Improving behavior, learning, and adjustment plays an important role in achieving better long-term outcomes for people with brain injuries (64). Psychosocial approaches such as CBT, group therapy, and support groups have been found useful in treating mental health concerns, teaching coping skills, and building strong social support systems. Comprehensive treatment plans tend to result in the most progress in emotional and social functioning (65).

A multimodal strategy—including methods like reward systems (contingency management), environmental adjustments (stimulus control), problem-solving exercises, and social skills training—can reduce aggression and improve behavior (66). In addition, cognitive rehabilitation also plays a key role. It uses techniques like direct and strategy training, metacognitive exercises, communication skills training, and memory aids to improve thinking and overall functioning (17).

### 2.7 Attention and communication rehabilitation

Attention process training and environmental modifications are effective in improving attention deficits. Additionally, communication rehabilitation through emotional perception and pragmatic skills training enhances social interactions and quality of life (67, 68). Rehabilitation of attention and communication skills is critical for individuals with TBI to regain functional independence and social participation. Attention deficits are common after TBI, significantly impacting cognitive and functional abilities. While conventional speech-language therapy addresses articulation and comprehension, specialized attention and communication rehabilitation focuses on executive communication skills, emotional perception, and pragmatic language. These are vital for social functioning and are often delivered through structured group programs or individualized cognitive-communication training (67, 68), can hinder social interaction and quality of life. Interventions to improve communication skills include speech therapy, language therapy, and social skills training.

Traumatic brain injury is a major global health concern, affecting approximately 64–74 million individuals worldwide annually (3). The effects of TBI can lead to motor and cognitive deficits (23, 69). Nutritional support and dietary protocols also play an important role in recovery following TBI (109). VR offers several advantages in rehabilitation, including enhanced motivation, real-time feedback, and the ability to simulate real-world scenarios.

### 2.8 Remote rehabilitation technologies

Remote technologies like wearable trackers and mobile apps are emerging as valuable tools, particularly for cognitive and motor rehabilitation. These technologies offer flexible, home-based solutions, though more research is needed to establish their long-term efficacy (117). Tele-rehabilitation programs utilize video conferencing and remote monitoring to provide therapy services, facilitating access to care and reducing healthcare costs, particularly beneficial for those in remote areas. Tele-ICU infrastructure may also be leveraged to manage several areas, including second opinions, imaging, stroke, and tele-counseling (70). Tele-monitoring can conserve resources by triaging remotely (71). The COVID era may benefit from remote rehabilitation. Combining traditional rehabilitation techniques with neuromodulation, biofeedback, and assistive devices enhances recovery. Personalized therapy based on patient and clinician needs maximizes neurorehabilitation outcomes (72).

## 3 Theoretical frameworks for rehabilitation

**Figure 4:**
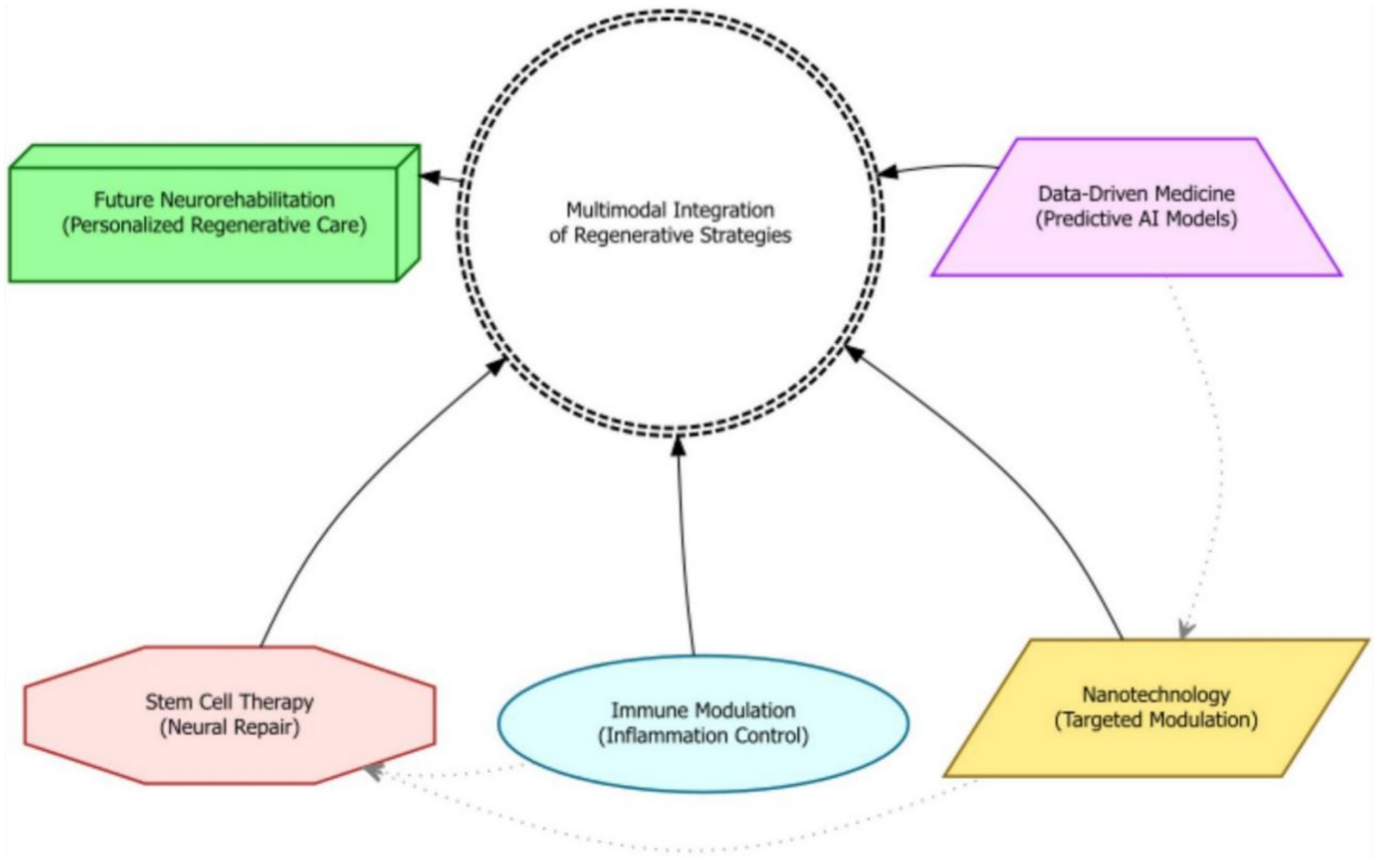
A hierarchical outcome model showing the progression from clinical indicators to long-term psychosocial wellbeing in moderate-to-severe TBI recovery. The framework emphasizes layered evaluation—from short-term recovery to reintegration and quality of life—culminating in clinically significant thresholds such as MDC and MCID.

Modern rehabilitative strategies are increasingly incorporating technological advancements, such as neuromodulation, biofeedback, and robotic devices, to enhance recovery outcomes (72). These technologies can augment traditional therapies by providing targeted stimulation, real-time feedback, and assistance with movement, thereby promoting neuroplasticity and functional restoration. Constraint-induced movement therapy, for example, has proven effective in improving upper extremity function by forcing the use of the affected limb. Robotic rehabilitation, with its capacity to deliver precise and repetitive movements, is opening new avenues for stroke rehabilitation and motor function restoration (73). Furthermore, recent studies suggest that advanced neurotechnological solutions, including robotic systems and electrodes that stimulate the nervous system, can enhance the effectiveness of stroke rehabilitation (74). The advent of virtual reality further enhances the rehabilitation landscape, offering immersive and interactive environments that simulate real-world scenarios, thereby facilitating motor and cognitive rehabilitation. Augmented reality systems also show promise in enhancing rehabilitation by providing real-time feedback and guidance during movement exercises. Ultimately, the judicious application of these technologies can significantly improve the precision and effectiveness of rehabilitation interventions, maximizing patient outcomes (Figure 4).

Stem cell-based therapies present a promising avenue for treating neurodegenerative disorders by potentially replacing damaged neural cells and stimulating endogenous repair mechanisms. When combined with nanotechnology, stem cell therapies can offer innovative strategies for improving stem cell delivery, modulating immune responses, and creating personalized regenerative treatments. Nanomaterials have been shown to stimulate tissue regeneration by delivering growth factors or drugs to specific brain regions, modulating inflammatory responses, and supporting neural cell growth and differentiation (3). Nanotechnology-enabled drug delivery systems allow for targeted and controlled release of therapeutics while minimizing off-target effects. Combining nanotechnology with stem cell therapies creates targeted and effective therapeutic interventions by using nanoparticles to deliver stem cell-specific factors or genetic material, promoting the survival, proliferation, and differentiation of transplanted stem cells. Additionally, data-driven approaches, utilizing vast amounts of patient data including imaging and genetic information, are being explored to identify optimal treatment strategies and predict individual patient responses. In the future, the convergence of nanotechnology and stem cell research may lead to personalized nanotherapeutics that deliver targeted agents to specific cells within the brain, enhancing therapeutic effects and minimizing side effects. Recent research also suggests that rehabilitation interventions can have direct regenerative effects, such as guiding stem cell differentiation, mobilizing stem cells into circulation, and enhancing the secretion of regenerative factors (75). Emerging areas of investigation, such as neuroimmune modulation, personalized therapeutics, and the combination of multiple nanotechnology-based strategies, hold immense promise for further enhancing the therapeutic potential of these innovative approaches. These strategies include the use of nanotechnology and neural stem cell niches to produce neurons, reprogramming local glial cells into neurons, and transplanting fetal progenitor cells (76).

Stem-cell–based therapies for TBI are promising in theory but face significant translational barriers. Clinical studies have shown that cell transplantation is generally safe and feasible, but efficacy has been limited (77). For example, trials of mesenchymal stromal cells (MSCs) and other stem cells report few adverse events, yet patient outcomes have not consistently improved (77). Major hurdles include ensuring safe and consistent cell production (e.g., standardized cell sources and dosing), achieving cell survival and integration in the injured brain, and avoiding risks such as immune reactions or tumorigenesis (77, 78). In animal models, genetically modified or immortalized cells improved recovery, but these raise “serious safety concerns” (especially cancer risk) when used in humans (78). Pluripotent stem cells (iPSCs) could provide patient-specific grafts, but are difficult and costly to produce and carry risks of ectopic cell growth (78). Overall, the complexity, cost, and ethical issues of stem cell production – combined with only modest clinical benefit so far – mean that translating stem cell therapies for TBI will require further research on optimal cell types, delivery methods, and long-term safety (77).

Nanotechnology approaches (e.g., nanoparticles for drug or gene delivery) also show promise but are in early stages of translation. The leaky blood–brain barrier after TBI offers opportunities for nanoparticle delivery, but systemic administration still faces challenges in targeting the brain while avoiding other organs (79). In practice, manufacturing and regulatory issues are major hurdles. Producing nanomedicines with consistent quality and safety (e.g., defined size, charge, biodegradability) is difficult at scale, and current regulatory pathways are not fully established for complex nanoformulations (80). Effective nanotherapies must balance brain uptake against toxicity elsewhere. For example, improving nanoparticle penetration through biological barriers (like the BBB) requires careful design of size and surface properties. As one recent review notes, “scale-up and manufacture of nanomaterials” is a key obstacle, and their FDA approval is complicated by multiple components (79). Although nanotechnology offers new tools (targeted drugs, imaging agents, scaffolds), advancing these to the clinic will demand overcoming delivery challenges, ensuring reproducible production, and early alignment with regulatory and manufacturing standards.

## 4 Evaluating rehabilitation efficacy

### 4.1 Outcome measures

Comprehensive assessment of rehabilitation in individuals with moderate-to-severe traumatic brain injury (TBI) requires using a variety of tools that measure different aspects of recovery. These tools help evaluate motor skills, thinking abilities, communication, emotional wellbeing, and the ability to perform daily tasks. Standardized tools like the Functional Independence Measure, the Disability Rating Scale, and the Glasgow Outcome Scale are commonly used to give measurable scores that reflect a person’s level of independence and disability.

Neuropsychological tests are important for checking problems in thinking, such as attention, memory, planning, and how fast a person can process information. These tests help guide treatment by identifying specific cognitive deficits. In addition to these standardized tests, using qualitative methods—like interviews with patients, input from caregivers, and observations—adds depth to the assessment by capturing the individual’s personal experience and how rehabilitation is affecting their life (Table 4).

**Table 4:** Key standardized and qualitative tools used to assess recovery outcomes in individuals with moderate-to-severe TBI.

Tool/scale	Domain assessed (cognitive, physical, emotional)	Type (standardized/qualitative)	Usage context	Limitations/benefits
Functional independence measure (FIM)	Physical, cognitive, daily living	Standardized	Assess overall independence in activities of daily living during and after rehab	Widely used and reliable but may not capture subtle cognitive/emotional changes
Disability rating scale (DRS)	Cognitive, physical, global disability	Standardized	Evaluate level of disability from coma to community reintegration	Good for broad disability levels but lacks detail on specific functions
Glasgow outcome scale (GOS)	Overall functional recovery	Standardized	Measure global outcomes post-TBI, often in clinical trials	Useful for long-term tracking but lacks sensitivity to nuanced progress
Neuropsychological tests	Cognitive (attention, memory, EF, processing speed)	Standardized	Identify specific cognitive deficits to tailor treatment and track progress	Highly informative but requires trained professionals and time to administer
Patient and caregiver interviews	Emotional, cognitive, quality of life	Qualitative	Gain personal insights into treatment impact and emotional wellbeing	Rich subjective data but may lack standardization and objectivity
Observational assessments	Physical, behavioral, cognitive performance	Qualitative	Assess functional abilities and recovery progress in real-world settings	Offers naturalistic data but can be influenced by observer bias

It outlines the domains each tool evaluates, how and when they are applied, and their relative strengths and limitations in clinical and real-world contexts.

It is also important to assess both short-term and long-term results of rehabilitation. This means tracking how a person improves during treatment and whether those improvements last over time. Long-term follow-up is essential to understand how effective the therapy has been and to identify what factors contribute to better recovery. Longitudinal studies, which observe patients over a longer period, are especially useful for learning about the lasting effects of treatment and what helps people achieve the best outcomes.

### 4.2 Statistical methodologies

Using strong statistical methods is important for analyzing data in rehabilitation research and making reliable conclusions about how well treatments work. Randomized controlled trials (RCTs) are considered the best way to evaluate the effectiveness of rehabilitation approaches. These studies use techniques like analysis of variance (ANOVA), *t*-tests, and regression analysis to compare results between different treatment groups (81). To understand how big the treatment effects are, researchers use measures like Cohen’s d and eta-squared. These help compare results across various studies and types of treatment (82).

Advanced methods like mediation and moderation analysis help explore how treatments work and which factors might affect the outcomes. These techniques are useful for understanding the deeper processes behind treatment success or failure. Using the right statistical tools helps make research stronger and supports better clinical decisions and healthcare policies (83). Stratifying patients—grouping them based on needs—is also important for targeting resources effectively (81). Tracking long-term treatment results is key to proving whether rehabilitation programs remain effective over time (84).

### 4.3 Clinical significance

**Figure 5:**
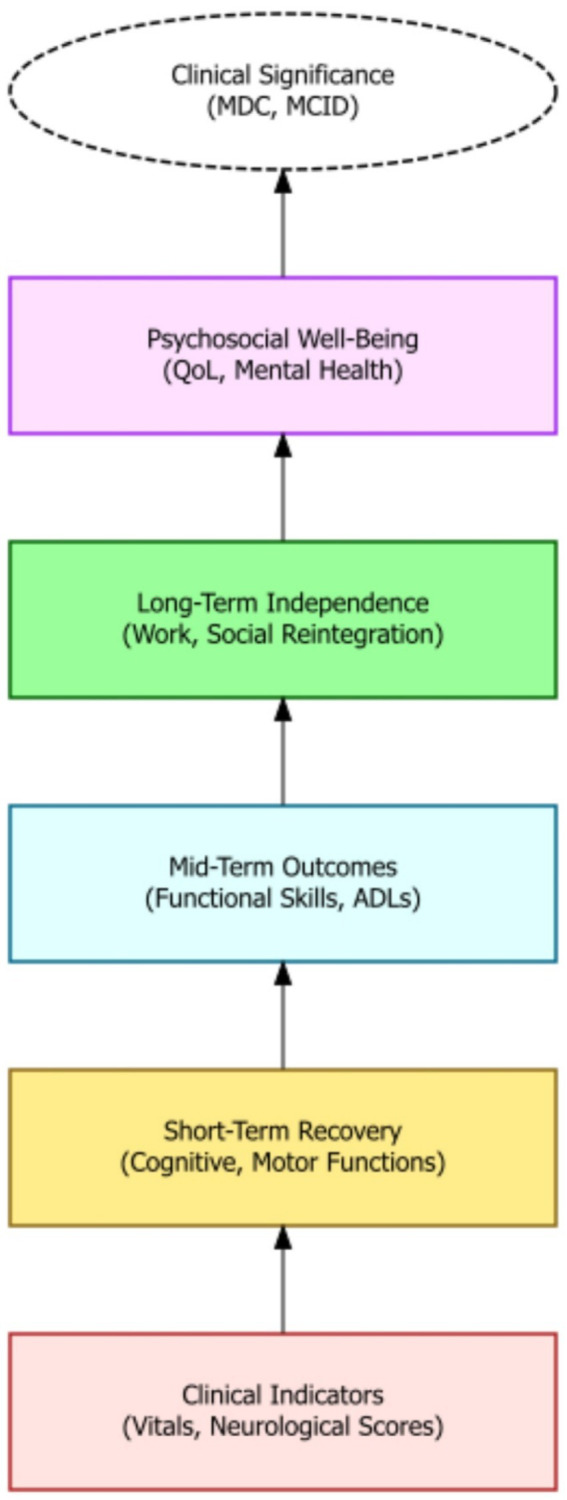
A schematic diagram depicting the convergence of advanced regenerative strategies—including stem cell therapy, nanotechnology, immune modulation, and data-driven medicine—into a multimodal integration hub for moderate-to-severe TBI rehabilitation.

Beyond statistical significance, the clinical significance of rehabilitation outcomes must be carefully considered to determine the practical relevance and impact of treatment effects. Clinically meaningful changes reflect functional or emotional improvements that are directly perceived by individuals with moderate-to-severe TBI and their families. Validated instruments such as the Patient-Reported Outcomes Measurement Information System (PROMIS), the Quality of Life after Brain Injury (QOLIBRI) scale, and the Canadian Occupational Performance Measure (COPM) are increasingly used to assess subjective improvements in health, daily functioning, and life satisfaction. Minimal detectable change and minimal clinically important difference values provide thresholds for determining whether observed changes in outcome measures represent clinically meaningful improvements. Qualitative research methods, such as interviews and focus groups, can provide rich contextual information about the lived experiences of individuals with TBI, informing the interpretation of quantitative outcome measures and identifying clinically relevant outcomes. The evaluation of clinical significance should consider the individual goals, values, and preferences of individuals with TBI, ensuring that treatment outcomes align with their priorities and enhance their quality of life (85). The integration of clinical expertise and patient perspectives is critical for guiding treatment. Shared decision-making (SDM) frameworks—where rehabilitation goals are co-developed by clinicians, patients, and families—have shown promise in aligning therapies with patient values. These models promote informed, value-based care and enhance treatment adherence, especially when supported by structured tools such as decision aids or outcome prioritization matrices (83, 85). Ultimately, rehabilitation should focus on improving function, activity and participation (Figure 5).

### 4.4 Evidence summary of clinical outcomes

#### 4.4.1 Telerehabilitation

Recent studies suggest that remote rehabilitation can yield outcomes comparable to in-person therapy for TBI. A 2018 systematic review of 13 studies (10 RCTs) found telephone-based interventions led to small-to-moderate benefits (effect size d = 0.28–0.51) in global functioning, mood, and sleep quality compared to usual care or wait-list controls, depending on study design (86). More recent meta-analytic evidence (17 RCTs, *N* = 3,158) indicates telehealth programs can modestly improve certain recovery metrics – for example, improving patients’ symptom self-management efficacy (standardized mean difference, SMD ≈ 0.22, 95% CI 0.02–0.42) – although effects on depression and neurobehavioral symptoms were not statistically significant (10). Notably, tele-cognitive training appears effective: one review reported that telerehabilitation produced greater gains in global cognitive function than wait-list/usual care and achieved non-inferior results to traditional in-clinic, therapist-led cognitive rehabilitation, which served as the active control condition in *mixed-severity TBI* and stroke populations (87). In a multicenter RCT on severe TBI, a telerehab program using a virtual reality home system led to significantly better functional independence (Barthel Index, *p* < 0.001) and executive function (Frontal Assessment Battery, *p* < 0.001) at follow-up than conventional rehab, along with large improvements in patient wellbeing (e.g., anxiety reduction, *ES*≈0.85) and caregiver burden relief (12). These findings underscore that telerehabilitation can effectively deliver TBI therapy, with measurable gains in functional and cognitive outcomes paralleling those of in-clinic rehabilitation (12, 87).

#### 4.4.2 Virtual reality (VR)–based interventions

Immersive and non-immersive VR therapies have shown promise for enhancing cognitive and motor recovery in patients with *moderate-to-severe TBI*. In one pilot trial, a VR-based driving simulation program for individuals with chronic mild-to-moderate TBI (defined as persistent post-concussion symptoms beyond 6 months) compared to a control group receiving therapist-led occupational therapy focused on cognitive compensation strategies and activities of daily living (ADL) simulations, which focused on cognitive compensation techniques and ADL simulation tasks (88), working memory (*p* = 0.004) and selective attention (*p* = 0.01) in the VR group versus a control group receiving standard occupational therapy (88). Likewise, VR cognitive training modules can yield broad neuropsychological benefits: a 2022 study of severe TBI patients reported significantly greater improvements in global cognition and attention for those receiving a non-immersive VR attention training program compared to a control group undergoing standard therapist-led attention and memory training using paper-and-pencil tasks (with additional gains noted in executive function and visuospatial skills on subtests) (89). The same pilot study by Andrei et al. (88) also reported that patients with chronic mild-to-moderate TBI improved on executive function (e.g., planning and problem-solving), with better coping and emotional outcomes, relative to a cognitive rehabilitation control group receiving therapist-led strategy training. Although not all trials find VR superior—particularly when compared to intensive therapist-led task-based training or computer-assisted therapy modules—the growing body of evidence suggests that VR interventions may boost specific cognitive domains such as attention, memory, and executive function, particularly when integrated with tailored therapy programs (90, 91). For instance, integrating VR into therapy has been associated with ~20–30% improvements on attention/executive function tests versus minimal changes with standard therapy (24), highlighting the potential of VR to engage patients and drive neuroplastic gains.

#### 4.4.3 AI and robotics

Advanced technologies like robotic-assisted training devices and AI-driven therapy aids have demonstrated quantifiable benefits in clinical TBI recovery. Robotic gait training systems (e.g., Lokomat) can deliver intensive motor therapy for individuals with moderate to severe TBI. One trial (88) found significant improvements in walking speed (self-selected gait velocity, *p* < 0.05) following robotic treadmill sessions compared to a control group that received manual physiotherapy and conventional gait training. In a comparative study, adding a VR module to robotic gait therapy yielded cognitive benefits beyond motor gains: only the group receiving combined Lokomat + VR showed significant post-treatment improvement in global cognitive function and executive attention (while both the VR and non-VR groups improved physical mobility and mood) (24). Robotics can also support upper-limb motor recovery in moderate-to-severe TBI, with case reports showing 10–20% improvements on validated motor function scales following robot-assisted training. However, these findings are typically uncontrolled or compared against historical baselines or standard upper-limb physiotherapy sessions (e.g., Fugl-Meyer Assessment) after robot-assisted training. The ± symbol has been corrected to reflect improvement ranges (10–20%). In addition, AI algorithms are being incorporated into rehabilitation robotics to personalize therapy. For example, an AI-enhanced humanoid robot (“Pepper”) equipped with a TBI-tailored facial emotion recognition model was able to detect patients’ emotional states with much higher accuracy (88% vs. 42% for standard software) (88), enabling more responsive interactive exercises. Early clinical use of such social robots in severe TBI has shown improved patient engagement and even psychosocial outcomes: a pilot RCT using a socially assistive robot reported greater gains in quality of life (an improvement ~1.5 points on EQ-5D index, *p* = 0.04) and larger reductions in depression (17.5-point drop on BDI-II by 3 months, *p* < 0.001) compared to conventional therapy (92). Overall, while research in TBI-specific robotics/AI is still emerging, these tools are demonstrating measurable enhancements in motor function, cognitive performance, and emotional wellbeing, suggesting they can augment traditional rehabilitation (12, 24, 88).

#### 4.4.4 Computerized cognitive rehabilitation

A growing body of RCTs and reviews indicates that computer-based training programs can significantly improve cognitive functions (such as attention, memory, processing speed, and executive skills) in TBI survivors. In a 2016 systematic review of 28 studies using computerized interventions for attention/executive deficits post-ABI, 23 studies (82%) reported significant gains in targeted cognitive outcomes after training (11). These interventions – often involving repetitive cognitive exercises or video game–like tasks – have been associated with measurable performance improvements: for example, patients completing a computerized cognitive training regimen have shown enhanced attention span and working memory (with medium effect sizes) relative to those receiving standard care or no treatment (11). Specific programs have yielded clinically relevant changes; one randomized trial of a structured computer-based cognitive training reported that twice as many TBI patients in the intervention group achieved large improvements compared to an active control group engaged in basic computer skills training unrelated to attention, memory, or executive function. Common benefits across studies include faster information processing, better memory recall, and improved executive function (planning, mental flexibility) – for instance, processing speed on timed tasks can improve by ~15–20% post-training in some programs, versus minimal change without intervention (93). Although methodological variations exist, the evidence supports computerized cognitive rehabilitation as an effective modality to boost cognitive recovery in individuals with mild-to-moderate TBI, particularly when targeting domains like attention and working memory that are critical for daily function (11) (118). In sum, leveraging computer-based drills and adaptive cognitive exercises enables intensive, individualized training that has been shown to translate into modest but significant improvements in real-world cognitive performance for TBI patients.

## 5 Discussion

Cognitive rehabilitation aims to improve cognitive functions such as attention, memory, executive function, and processing speed. Evidence suggests that cognitive rehabilitation can enhance cognitive abilities and improve functional outcomes in individuals with mild-to-moderate TBI (94). Specific cognitive rehabilitation techniques, such as attention training, memory strategies, and problem-solving training, have demonstrated efficacy in improving cognitive performance and promoting generalization to real-world settings. Computerized cognitive rehabilitation interventions have shown promise in enhancing attention and executive functioning after acquired brain injury; however, their implementation faces several challenges. These include a lack of standardized treatment protocols, limited consensus on therapy intensity and duration, difficulties in tailoring programs to individual cognitive profiles, and a shortage of interdisciplinary guidelines to align digital tools with clinical decision-making (11). Scalability is also a concern, especially in low-resource settings where access to technology and trained facilitators may be limited. Virtual reality-enhanced rehabilitation shows moderate promise in improving cognitive deficits, particularly among individuals with mild-to-moderate TBI (7). However, the effectiveness of cognitive rehabilitation may vary depending on the severity and nature of cognitive deficits, as well as individual factors such as motivation, learning style, and psychosocial support. Future research should explore the long-term effects of cognitive rehabilitation and identify predictors of treatment response (95, 110).

For example, Hallock et al. (83) found significant gains in executive functioning (Hedges’ g = 0.48, *p* < 0.01) following computerized cognitive rehabilitation in TBI patients. Similarly, Calabrò et al. (12) demonstrated that a telerehabilitation program incorporating virtual reality significantly improved patients’ functional independence and executive function (*p* < 0.001), along with large reductions in anxiety symptoms (effect size ≈ 0.85). These outcomes underscore the importance of integrating data-driven and technology-assisted methods into neurorehabilitation. While traditional therapies remain vital, evidence now supports supplementing them with emerging modalities—particularly for enhancing cognitive performance, executive skills, and daily functioning post-TBI.

Physical and motor rehabilitation focuses on improving motor skills, balance, coordination, and mobility in individuals with moderate-to-severe TBI. Rehabilitation interventions, such as gait training, balance exercises, and constraint-induced movement therapy, have demonstrated effectiveness in improving motor function and reducing disability after TBI (96). Task-oriented training, which involves practicing functional tasks in real-world contexts, has been shown to enhance motor learning and promote transfer of skills to everyday activities. Assistive devices, such as orthotics, wheelchairs, and adaptive equipment, can improve mobility, independence, and participation in individuals with motor impairments. The amount and intensity of exercise, personal implication and/or determination, and task-oriented training play a crucial role in the outcome (97). Robot-assisted therapy can provide repetitive, high-intensity training and improve upper limb motor function after stroke (98). Further research is needed to determine the optimal timing, intensity, and duration of physical and motor rehabilitation interventions, as well as to identify factors that predict treatment response.

Speech and language rehabilitation aims to improve communication skills, including speech production, language comprehension, and social communication, in individuals with TBI. Speech therapy interventions, such as articulation therapy, language therapy, and augmentative and alternative communication strategies, have demonstrated effectiveness in improving communication abilities and reducing communication barriers after TBI. Group therapy and peer support can provide opportunities for individuals with communication disorders to practice communication skills in a supportive and naturalistic environment (99). Tele-neurorehabilitation is effective for patients who had glioblastoma (100). The efficacy of speech and language rehabilitation may vary depending on the type and severity of communication deficits, as well as individual factors such as cognitive abilities, motivation, and social support. Future research should investigate the effectiveness of different speech and language rehabilitation approaches for specific communication disorders after TBI, as well as the impact of communication interventions on social participation and quality of life.

Psychological and emotional rehabilitation addresses the psychological, emotional, and behavioral challenges that individuals with TBI may experience, such as depression, anxiety, post-traumatic stress disorder, and aggression. Cognitive-behavioral therapy, mindfulness-based interventions, and psychotherapy have demonstrated efficacy in reducing psychological distress and improving emotional wellbeing after TBI. Support groups and peer mentoring can provide emotional support, reduce isolation, and promote resilience in individuals with psychological and emotional difficulties. Pharmacological interventions, such as antidepressants and anti-anxiety medications, may be used in conjunction with psychological therapies to manage mood and anxiety symptoms after TBI. Addressing psychological issues that arise post TBI are important, since some patients visit a psychiatrist several years post trauma (101). Further research is needed to determine the optimal combination of psychological and pharmacological interventions for managing psychological and emotional sequelae after TBI, as well as to identify factors that predict treatment response and long-term outcomes.

At the same time, it is noteworthy that these diverse rehabilitation approaches – telerehabilitation, VR, robotics/AI, and computer-based training – all demonstrate quantitative benefits for TBI recovery, often comparable to or exceeding conventional therapy on specific outcomes. This convergence of evidence suggests that integrating technology-assisted interventions can enhance TBI rehabilitation by extending the reach and intensity of therapy without compromising efficacy. For example, the finding that remote and VR-assisted therapies can match in-person treatment outcomes (87) underscores a practical advantage: patients who face barriers to on-site rehab can still achieve meaningful improvements through telehealth and home-based programs. Likewise, the augmentative effects of VR and robotic systems (e.g., boosting executive function or gait speed beyond standard care alone) highlight their role as valuable complements to traditional rehabilitation. However, the range of effect sizes observed (from small gains in some telerehab trials to large improvements in targeted domains with VR or robotics) also points to the importance of individualized therapy plans – selecting the right combination of conventional and innovative modalities to address each patient’s unique deficits. In sum, the clinical outcome evidence supports a multimodal rehabilitation strategy in TBI: one that harnesses emerging technologies to maximize functional and cognitive recovery, while continuing to build on the foundation of evidence-based therapeutic principles established in conventional care.

### 5.1 Research gaps and future directions

Despite the advances in rehabilitation approaches for TBI, several gaps remain in the research literature that warrant further investigation. First, more research is needed to understand the long-term effects of rehabilitation interventions on functional outcomes, quality of life, and community reintegration after TBI. Second, there is a need for more studies that examine the effectiveness of multidisciplinary rehabilitation approaches that integrate cognitive, physical, speech, and psychological therapies to address the complex and multifaceted needs of individuals with TBI. Third, future studies should employ rigorous research designs, such as randomized controlled trials, to evaluate the efficacy of rehabilitation interventions and minimize bias. Fourth, there is a need for more research that focuses on identifying predictors of treatment response and tailoring rehabilitation interventions to individual patient characteristics and needs (102). Fifth, it is important to investigate the cost-effectiveness of different rehabilitation approaches and to develop strategies for optimizing resource allocation and access to care for individuals with TBI. Lastly, mild TBIs are highly comorbid with posttraumatic stress disorder, which represents a potential complicating factor in recovery (103, 104). Individuals with comorbid posttraumatic stress disorder and TBI have significantly poorer clinical outcomes than individuals (105, 111) (Table 5).

**Table 5:** Key gaps in moderate-to-severe traumatic brain injury (TBI) rehabilitation research, their impact on clinical outcomes and research validity, and provides evidence-based recommendations for future investigation.

Identified gap	Impact on field	Suggested future work	References
Limited understanding of long-term effects of rehabilitation on TBI outcomes	Uncertainty about sustainability of treatment effects; limits evidence-based practice	Conduct longitudinal studies assessing functional, cognitive, and social outcomes over time	Glenn (102) and Rees et al. (95)
Lack of studies integrating multidisciplinary rehabilitation approaches	Incomplete treatment strategies that fail to address complex patient needs	Design and evaluate integrated rehabilitation models combining physical, cognitive, and emotional therapies	Cicerone et al. (94) and Afsar et al. (17)
Insufficient use of rigorous research designs in TBI rehabilitation studies	Potential bias and reduced validity of findings; weaker clinical recommendations	Increase randomized controlled trials and use standardized outcome measures	Hallock et al. (83) and Morone and Pichioori (81)
Need for patient-specific rehabilitation approaches based on individual characteristics	Generic treatments may result in suboptimal outcomes and reduced patient engagement	Identify predictors of treatment response to tailor therapies to patient profiles	Rees et al. (95) and Kim (38)
Limited data on cost-effectiveness and resource allocation for rehabilitation	Inefficient healthcare planning and reduced accessibility for patients	Evaluate economic aspects of rehabilitation to improve policy and funding decisions	Samuelson et al. (103) and Vasterling et al. (104)
High comorbidity of TBI with PTSD and its impact on recovery	Overlooks compounded psychological effects and worsened patient outcomes	Investigate tailored treatment protocols for TBI-PTSD comorbid patients	Weis et al. (105)
Challenges in translating nanotechnology and stem cell therapies into clinical practice	Delays in developing advanced, personalized therapies for neuroregeneration	Focus on safety, delivery mechanisms, and personalization of regenerative treatments	Deng et al. (75) and Clark et al. (76)

The convergence of nanotechnology and stem cell-based therapies heralds a transformative era in regenerative medicine, especially for neurodegenerative disorders. While this integration shows promise, the safety and biocompatibility must be carefully assessed to ensure effective translation into clinical practice. Ongoing studies are enhancing stem cell delivery, improving survival and engraftment, and modulating immune responses for effective tissue regeneration. Future research should focus on addressing current limitations such as overcoming the blood–brain barrier, ensuring targeted therapeutic delivery, and optimizing stem cell integration. Personalized nanotherapeutics, tailored to individual patient needs, represent a significant stride in this direction. Further investigations into neuroimmune modulation and combinatorial nanotechnology strategies could amplify the therapeutic potential, paving the way for revolutionary treatments for neurodegenerative conditions. The development of such therapies requires multidisciplinary collaboration among experts in nanoscience, stem cell biology, neuroscience, immunology, and clinical medicine. Moreover, emphasis should be placed on applying this new methodology to the difficult, complex clinical scenarios of combined traumatic injuries and especially integrating additional factors such as age, gender, genetics, and co-morbidities (106). Ultimately, personalized medicine approaches, leveraging patient-derived stem cells and nanotechnology, hold immense promise for customized treatments tailored to individual genetic and molecular profiles, improving both efficacy and safety. The utilization of near-infrared spectroscopy allows for high-temporal resolution cerebral physiome characterization in patients with TBI, which could lead to more targeted therapeutic strategies and precision medicine (107). Every neurovascular event is unique in terms of location, severity and affected systems, and with emerging knowledge on neural plasticity and improvements in stimulation techniques and protocols, the upcoming decades may lead to highly effective interventions to drive neuronal reorganization and functional recovery.

To further advance clinical outcomes in traumatic brain injury rehabilitation, future research must also explore personalized monitoring tools and regenerative science. Recent work by Gomez et al. (107) shows how near-infrared spectroscopy (NIRS) can provide real-time, high-temporal resolution insights into cerebral oxygenation and hemodynamics. Such technologies allow clinicians to fine-tune interventions based on dynamic physiological markers, potentially enabling more precise and individualized rehabilitation strategies. Similarly, regenerative rehabilitation—a multidisciplinary field that combines physical therapy with biological interventions such as stem cell therapy and nanotechnology—is emerging as a promising area for enhancing neuroplasticity and tissue repair (75). Integrating these approaches with traditional rehabilitation holds the potential to revolutionize how recovery is supported at the cellular and functional level in TBI survivors.

## 6 Conclusion

In practice, effective TBI rehabilitation relies on multidisciplinary care: clinicians (neurologists, physiatrists, therapists, neuropsychologists, etc.) must collaborate to address each patient’s unique deficits. The chronic impact of TBI on functional outcomes and quality of life must be considered throughout the rehabilitation process (112). Rehabilitation programs should be individualized and neuropsychology-informed – for example, tailoring therapy to the patient’s cognitive profile, emotional status, and personal goals. Recent reviews stress that neuropsychological interventions must be adapted to each patient’s context (considering injury severity, timing, and symptoms) to be effective. Training and involving neuropsychologists in rehab teams can improve outcomes by optimizing cognitive and behavioral interventions. Looking ahead, research should prioritize integrated, patient-centered approaches. This includes developing coordinated interdisciplinary protocols (combining physical therapy, cognitive rehab, counseling, etc.) and leveraging biomarkers or neuroimaging to personalize treatment. Large-scale collaboration (clinicians, researchers, patients) will be needed to conduct rigorous trials and to translate promising therapies (like stem cells or neuromodulation) into practice. The rehabilitation interventions must be applied in a coordinated, personalized way to reduce long-term disability, and should call for future studies on interdisciplinary care models and personalized neurorehabilitation strategies.
