# Correction: A comprehensive review of rehabilitation approaches for traumatic brain injury: efficacy and outcomes

**DOI:** 10.3389/fneur.2025.1651979

**Published:** 2025-07-08

**Authors:** Ye Shen, Linzhi Jiang, Junmei Lai, Jiahui Hu, Feng Liang, Xingru Zhang, Fangfang Ma

**Affiliations:** ^1^Department of Rehabilitation Medicine, Zhejiang Provincial People's Hospital, People's Hospital of Hangzhou Medical College, Hangzhou, China; ^2^Geriatric Medicine Center, Department of Geriatric Medicine, Department of Nursing, Zhejiang Provincial People's Hospital (Affiliated People's Hospital), Hangzhou Medical College, Hangzhou, Zhejiang, China

**Keywords:** traumatic brain injury, traumatic brain injury rehabilitation, cognitive therapy, telerehabilitation, medical trends

In the published article, there was an incorrect **Funding** statement. The correct funding statement is:

**Funding**

The author(s) declare that financial support was received for the research and/or publication of this article. This study was supported by grants from the Zhejiang Medical and Health Science and Technology Program (no. 2025KY021).

The original version of this article has been updated.

